# Have we missed that neural vasodilator mechanisms may contribute to exercise hyperemia at onset of voluntary exercise?

**DOI:** 10.3389/fphys.2013.00023

**Published:** 2013-02-15

**Authors:** Kanji Matsukawa, Kei Ishii, Nan Liang, Kana Endo

**Affiliations:** Department of Integrative Physiology, Graduate School of Biomedical and Health Sciences, Hiroshima UniversityMinami-ku, Hiroshima, Japan

**Keywords:** central command, exercise, vasodilatation, skeletal muscle, near infrared spectroscopy

## Abstract

Whether neurally-mediated vasodilatation may contribute to exercise hyperemia has not been completely understood. Bülbring and Burn (1935) found for the first time the existence of sympathetic cholinergic nerve to skeletal muscle contributing to vasodilatation in animals. Blair et al. (1959) reported that atropine-sensitive vasodilatation in skeletal muscle appeared during arousal behavior or mental stress in humans. However, such sympathetic vasodilator mechanism for muscle vascular bed in humans is generally denied at present, because surgical sympathectomy, autonomic blockade, and local anesthesia of sympathetic nerves cause no substantial influence on vasodilatation in muscle not only during mental stress but also during exercise. On the other hand, neural mechanisms may play an important role in regulating blood flow to non-contracting muscle. Careful consideration of the neural mechanisms may lead us to an insight about a possible neural mechanism responsible for exercise hyperemia in contracting muscle. Referring to our recent study measuring muscle tissue blood flow with higher time resolution, this review has focused on whether or not central command may transmit vasodilator signal to skeletal muscle especially at the onset of voluntary exercise.

## Introduction

Bülbring and Burn (1935) reported for the first time the existence of sympathetic cholinergic nerve to skeletal muscle contributing to vasodilatation in the cat and dog. It is histologically verified that sympathetic cholinergic nerves innervate blood vessels of skeletal muscle in several species such as the cat, dog, sheep, etc., while muscle vasculature in the rat and mouse lacks sympathetic cholinergic innervation (Bolme and Fuxe, 1970; Burnstock, 1980; Guidry and Landis, 2000). Moreover, there is no histological evidence for the existence of sympathetic cholinergic nerves to skeletal muscle in the monkey and man (Bolme and Fuxe, 1970). Atropine-sensitive vasodilatation in skeletal muscle occurs during fighting or arousal behavior in cats and dogs (Ellison and Zanchetti, 1973; Just et al., 2000). Although Blair et al. (1959) reported that the forearm vasodilator response to mental stress in humans was atropine-sensitive and became absent after surgical sympathectomy, no electrophysiological evidence for sympathetic cholinergic nerves has been demonstrated in humans (Wallin and Sundlöf, 1982; Saito et al., 1993; Callister et al., 1994). Joyner and Dietz (2003) suggested that circulating epinephrine and locally released acetylcholine, but not sympathetic dilator nerve, play a role in producing the vasodilator response to mental stress in humans. Even in the dog possessing the sympathetic cholinergic system, exercise hyperemia of hindlimb contracting muscle is not significantly influenced by surgical sympathectomy or ganglionic or muscarinic blockade (Donald et al., 1970; Buckwalter et al., 1997; Buckwalter and Clifford, 1999). Taken together, it has been currently thought that the sympathetic nervous system is not responsible for exercise hyperemia as well as vasodilation during mental stress, although sympathetic vasoconstriction restrains blood flow to active muscle during exercise (Joyner et al., 1992; Joyner and Halliwill, 2000; Clifford and Hellsten, 2004; Joyner and Wilkins, 2007; Shoemaker, 2012). Since most previous findings were obtained with measurements of limb blood flow via venous occlusion plethysmography and Doppler ultrasound at relatively low time resolution, a possible contribution of neurally-mediated vasodilatation to exercise hyperemia will be discussed referring to a recent study which measured muscle tissue blood flow with near-infrared spectroscopy (NIRS) at higher time resolution (Ishii et al., 2012).

## Sympathetic vasodilatation in skeletal muscle during exercise in animals

Sympathetic cholinergic nerve can be activated when the localized areas in the hypothalamus, the midbrain periaqueductal gray matter, and the midbrain ventral tegmental area (VTA) are stimulated in the cat (Eliasson et al., 1951; Abrahams et al., 1960; Hilton et al., 1983; Bandler and Carrive, 1988; Matsukawa et al., 1993, 2011). It is of interest that electrical and chemical stimulation of neurons in the VTA increased blood flow and vascular conductance of the rat triceps surae muscle (Matsukawa et al., 2011; Nakamoto et al., 2011), although its muscular vasculature lacks sympathetic cholinergic innervation (Guidry and Landis, 2000). The vasodilation may be mediated by nitrosyl factors released from sympathetic postganglionic fibers and/or the vascular endothelium (Davisson et al., 1994). When visualizing using an X-ray angiography the vascular responses of small arteries (internal diameter, 100–500 μm) in the cat triceps surae muscle, stimulation of the hypothalamic defense area caused tremendous increases in internal diameter and cross sectional area of the small arteries, which were abolished by muscarinic blockade or section of the sciatic nerve (Matsukawa et al., 1997). Although the vasodilation is mediated by activating sympathetic cholinergic nerve, whether the sympathetic cholinergic system is functionally operating during exercise is controversial.

**Figure 1:**
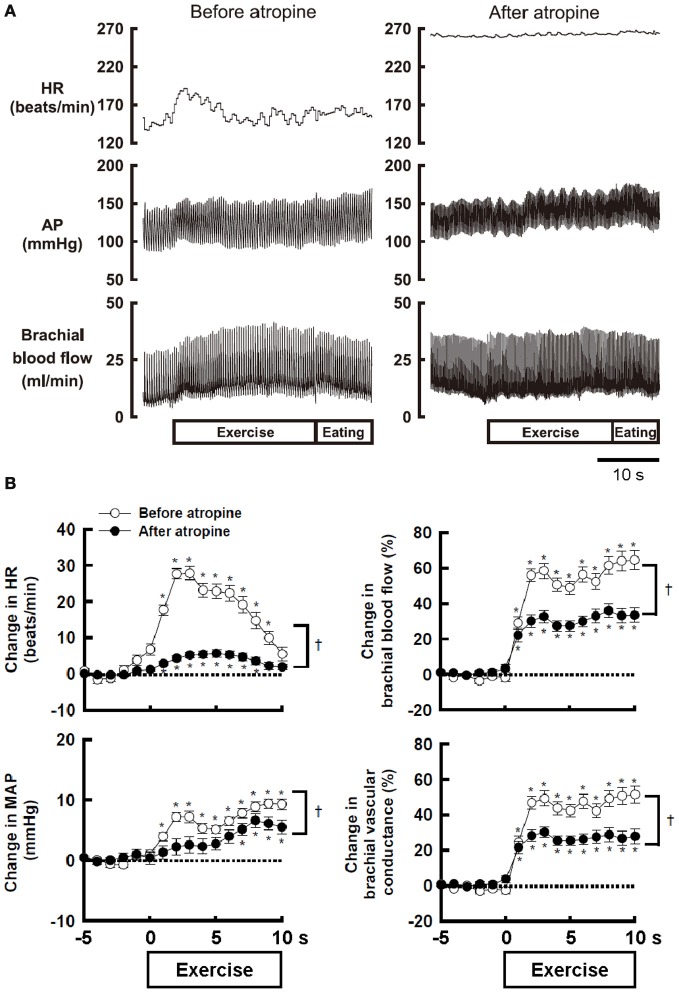
**(A)** The effect of atropine on the responses in heart rate (HR), arterial blood pressure (AP), and brachial blood flow during voluntary static exercise in a conscious cat. In the presence of atropine, the increase in brachial blood flow was blunted, although the baseline blood flow was not altered. **(B)** The effect of atropine on the average responses in HR, mean arterial blood pressure (MAP), brachial blood flow, and brachial vascular conductance during static exercise. All cardiovascular responses at the onset of exercise were blunted by atropine. Especially, the peak increases in brachial blood flow and vascular conductance were decreased by atropine to 54–55% of the control responses. ^†^Significant difference (*P* < 0.05) before and after atropine. ^*^Significant difference (*P* < 0.05) at a given time from the preexercise level. Adopted from Komine et al. (2008) with permission.

Atropine-sensitive vasodilatation in skeletal muscle occurs during fighting or arousal behavior or classical conditioning of limb flexion with conditioned audio-tone stimulus and unconditioned electrical stimulation of a paw in animals (Ellison and Zanchetti, 1973; Just et al., 2000). However, the increase in femoral blood flow during treadmill exercise is unaffected by surgical sympathectomy or muscarinic blockade (Donald et al., 1970; Buckwalter et al., 1997; Buckwalter and Clifford, 1999). A reason responsible for the discrepancy may be that the sympathetic cholinergic vasodilatation, if any, may be masked by other mechanisms such as metabolic or flow-mediated vasodilatation and may appear during a voluntary type of exercise with a smaller muscle mass, rather than automatic rhythmic movement with whole body mass. Komine et al. (2008) found that brachial blood flow of the exercising forelimb, heart rate (HR), arterial blood pressure (AP) increases at the onset of voluntary static exercise in cats and intravenous injection of atropine blunts the increases in brachial blood flow and vascular conductance (Figure 1). It is likely that the sympathetic cholinergic mechanism is capable of evoking muscle vasodilatation at the onset of a voluntary type of exercise in conscious animals as well as fighting or arousal behavior.

## Muscle sympathetic nerve activity during exercise in humans

Sympathetic mechanisms responsible for muscle vasodilatation, if any, can be explained by withdrawal of sympathetic adrenergic vasoconstrictor activity and/or facilitation of sympathetic cholinergic or nitroxidergic vasodilator activity. Saito et al. (1993) and Callister et al. (1994) reported that muscle sympathetic nerve activity (MSNA) to a resting leg or arm decreases during anticipation and initiation of cycling exercise and then increases during the later period of exercise, suggesting sympathetic withdrawal prior to and at the start of exercise. However, the response of MSNA to non-contracting muscle at the onset of one-legged cycling is still controversial [increased (Herr et al., 1999), decreased (Saito and Mano, 1991), and unchanged (Ray et al., 1993)]. Fisher et al. (2005) reported that vascular conductance in the resting leg transiently increases at the onset of contralateral isometric exercise, whereas MSNA to the leg is unchanged, suggesting that the transient increase in vascular conductance at the onset of exercise is unrelated to the changes in MSNA. Thus, withdrawal of muscle sympathetic vasoconstrictor activity if any cannot explain the initial vasodilatation during exercise.

Regarding sympathetic cholinergic nerve in animals, only a few studies have reported activity of presumable muscle sympathetic cholinergic fibers in the cat (Horeyseck et al., 1976; Lopes and Palmer, 1977; Dean and Coote, 1986). These studies revealed that postganglionic cholinergic fibers have quite different characteristics from sympathetic adrenergic vasoconstrictor fibers and they are spontaneously inactive and excited in association with atropine-sensitive vasodilatation during hypothalamic stimulation. In contrast, a microneurographical attempt to record sympathetic cholinergic vasodilator activity has been failed in humans (Wallin and Sundlöf, 1982; Saito et al., 1993; Callister et al., 1994). This may be attributed to no anatomical innervation of sympathetic cholinergic nerve for skeletal muscle (Bolme and Fuxe, 1970) or to a reason that it is difficult to measure sympathetic cholinergic activity with a conventional microelectrode. Thus, direct electrophysiological evidence for sympathetic cholinergic nerves is lacking in humans.

## Can neural mechanisms contribute to exercise hyperemia in skeletal muscle at the onset of exercise in humans?

**Figure 2:**
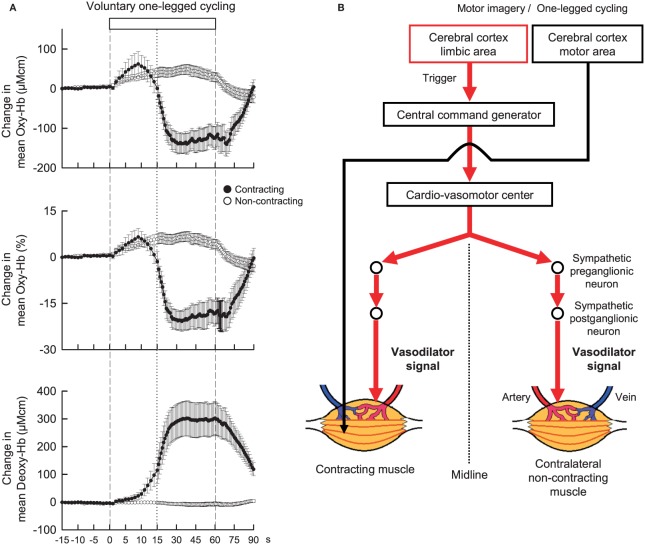
**(A)** The time courses of the relative changes in oxygenated-hemoglobin (Oxy-Hb) and deoxygenated-hemoglobin (Deoxy-Hb) of the contracting (•) and non-contracting (◦) vastus lateralis (VL) muscles during voluntary one-legged cycling exercise in humans. Vertical dashed lines indicate the start and end of one-legged cycling. A vertical dotted line is placed at 15 s from the exercise onset. At the start of voluntary one-legged exercise, Oxy-Hb in the contracting and non-contracting VL muscles increased with the same time course and magnitude. There was no significant difference (*P* > 0.05) in the Oxy-Hb response between the two muscles at the initial 15 s period of exercise. Subsequently, Oxy-Hb decreased and Deoxy-Hb increased in the contracting VL as long as exercise was continued. Adopted from Ishii et al. (2012) with permission. **(B)** A hypothesis that centrally-induced vasodilator signal is equally transmitted to bilateral VL muscles at the start of voluntary one-legged exercise in humans.

Neural mechanisms may play an important role in regulating blood flow in non-contracting muscle. Accordingly, before considering neural mechanisms responsible for exercise hyperemia in contracting muscle, neural control of blood flow in non-contracting muscle during contralateral limb exercise should be taken into account. As candidates for this, it is considered that sympathetic cholinergic and/or β-adrenergic mechanisms may cause vasodilation in blood vessels of in non-contracting muscle, while a sympathetic α-adrenergic mechanism may cause vasoconstriction (Eklund and Kaijser, 1976; Sanders et al., 1989; Reed et al., 2000). These mechanisms affect in concert blood flow to non-contracting muscle, depending on the time course, modality, and intensity of exercise, to what extent muscle mass is engaged during exercise, of which limb blood flow is measured, and age (Duprez et al., 1989; Taylor et al., 1989, 1992). For example, the vascular response of non-contracting muscle changes along the time course of exercise (Taylor et al., 1989). However, as long as the initial transient phase of exercise is targeted, blood flow in a resting limb may increase during static or dynamic exercise of the contralateral limb (Eklund et al., 1974; Eklund and Kaijser, 1976; Rusch et al., 1981; Taylor et al., 1989; Jacobsen et al., 1994). Eklund and Kaijser (1976) suggested that vasodilatation of the resting forearm during contralateral handgrip is mediated by β-adrenergic mechanisms and, if the contraction is prolonged, α-adrenergic vasoconstriction takes place. Reed et al. (2000) suggested that β-adrenergic mechanisms due to circulating catecholamines and locally-released nitric oxide contribute to the vasodilatation, while sympathetic dilator nerves are not responsible for the limb vasodilatation seen after stellate block. In contrast, Sanders et al. (1989) reported that the initial vasodilatation in the resting limb was blocked by atropine but not by propranolol, suggesting that sympathetic cholinergic nerves may play a role in causing vasodilatation. The discrepancy among the previous findings may be partly attributed to technical limitation of blood flow measurement with venous occlusion plethysmography, which is a useful technique but does not provide the rapid dynamic changes in muscle tissue blood flow (Casey et al., 2008). Recently, Ishii et al. (2012) examined the dynamic changes in concentration of oxygenated-hemoglobin (Oxy-Hb) of the non-contracting vastus lateralis (VL) muscle with NIRS as an index of muscle tissue blood flow. The Oxy-Hb in the non-contracting VL rapidly increased at the start of contralateral one-legged exercise (Figure 2A) but failed to increase at the start period of passive one-legged exercise. Since the Oxy-Hb also increased during mental imagery of the exercise, central command may contribute to increasing tissue blood flow in the non-contracting muscle at the start of contralateral exercise. On the other hand, Fisher and White (2003) reported that both voluntary and electrically-evoked isometric plantar flexion caused an initial increase in calf vascular conductance of the contralateral resting leg and Wray et al. (2005) and Trinity et al. (2010, 2011) reported that passive knee movement increased blood flow to the contralateral leg. These studies suggest that exercise pressor reflex, probably muscle mechanoreflex, may play a role in inducing the contralateral vasodilatation as well. In addition, the hyperemic response may result in part from a central hemodynamic mechanism, i.e., an increase in cardiac output resultant from mechanoreflexly evoked tachycardia (Trinity et al., 2010, 2011).

Control of increased blood flow to active muscles is much more complicated and is mainly the result of the interplay of neural vasoconstrictor activity, locally derived vasoactive substances (released from contracting muscles, vascular endothelium, or red blood cells), and mechanical factors (Rådegran and Saltin, 1998; Saltin et al., 1998; Clifford and Hellsten, 2004; Wray et al., 2005; Trinity et al., 2010, 2011, 2012). On the other hand, it has been thought that the sympathetic nervous system does not appear to be responsible for vasodilatation, although sympathetic α-adrenergic vasoconstrictor restrains blood flow to active muscles during exercise (Williams et al., 1985; Joyner et al., 1992; Clifford and Hellsten, 2004; Joyner and Wilkins, 2007). Instead, locally derived vasoactive substances and mechanical factors should determine the increase in blood flow to active muscle. Nevertheless, since the increase in Oxy-Hb of the contracting muscle had the almost same time course and magnitude as the increase in Oxy-Hb of the non-contracting muscle at the initial 15-s period of one-legged cycling (Figure 2A; Ishii et al., 2012), this finding leads to an idea that centrally-induced vasodilator signal may be transmitted to active muscle at least partly at the start of exercise (Figure 2B). However, a more comprehensive study including autonomic blockade will be necessary to test this hypothesis.

### Conflict of interest statement

The authors declare that the research was conducted in the absence of any commercial or financial relationships that could be construed as a potential conflict of interest.
